# Efficacy of antibiotherapy for treating flatus incontinence associated with small intestinal bacterial overgrowth: A pilot randomized trial

**DOI:** 10.1371/journal.pone.0180835

**Published:** 2017-08-01

**Authors:** Chloé Melchior, Guillaume Gourcerol, Valérie Bridoux, Philippe Ducrotté, Jean-François Quinton, Anne-Marie Leroi

**Affiliations:** 1 INSERM U1073, Service de Physiologie Digestive, CHU Rouen, INSERM CIC 0204 Rouen, Rouen, France; 2 INSERM U1073, Service de Chirurgie Digestive, CHU Rouen, Rouen, France; 3 INSERM U1073, Service d'Hépato-Gastroentérologie, CHU Rouen, Rouen, France; 4 Service d’Hépato-Gastroentérologie, CHU Lille, Lille, France; Stavanger University Hospital, NORWAY

## Abstract

**Aim:**

An increase in intestinal gas production due to small intestinal bowel overgrowth (SIBO) is a contributing factor for flatus incontinence. The aims of our study were to assess the efficacy of metronidazole in a select population of patients with flatus incontinence associated with SIBO and to compare its efficacy with that of a combination of simethicone and activated charcoal (SC; Carbosylane) in randomized experimental arms.

**Methods:**

Adult patients suffering from flatus incontinence associated with SIBO diagnosed by a glucose breath test were enrolled in the study. They were given metronidazole or Carbosylane (SC) for 10 days. The reduction in the mean daily number of gas leakages reported in a 3-day diary before and at the end of the treatment was used as the primary endpoint.

**Results:**

Of 52 consecutive subjects with flatus incontinence, 23 (44%) had SIBO, 16 (33%) of whom were included in and completed the study. The relative reduction in flatus incontinence episodes was significantly higher in the metronidazole than in the SC group (66.8±34.8% vs. 25±50%, P = 0.03), decreasing by more than 50% in 7 (87.5%) of the subjects in the metronidazole group compared with only 1 (12.5%) in the SC group (odds ratio 1.9, 95% confidence interval 0.9–56.9, P = 0.06).

**Conclusion:**

Our results show a promising trend indicating that metronidazole might significantly improve flatus incontinence associated with SIBO and might be more successful in treating flatus incontinence than gas absorbents.

## Introduction

In most patients, involuntary leakage of gas through the anus is limited to flatus incontinence, although this type of incontinence is occasionally associated with liquid or solid stool incontinence. Flatus incontinence is the most common type of anal incontinence, with a prevalence estimated at approximately 33% of the general adult population [1]. While it appears to have less impact upon quality of life than fecal incontinence [2], it remains an embarrassing and socially restricting problem [3–4] due to the poor efficacy of the therapeutic options, which are mainly restricted to gas absorbents. Although the mechanism of flatus incontinence is poorly understood, increased intestinal gas production is likely involved.

Small intestine bacterial overgrowth (SIBO), which is associated with increased intestinal gas production, is defined as the presence of abnormal and excessive numbers of bacteria in the small intestine [5]. It is characterized a myriad of symptoms, including bloating, flatulence, abdominal pain, nausea, dyspepsia, diarrhea, and constipation [6]. The presence of at least 10^5^ CFU/mL of bacteria in jejunal aspiration cultures is considered the gold standard for diagnosing SIBO. However, hydrogen and/or methane breath tests are commonly used in clinical practice since they are more acceptable to patients and provide quicker results for clinicians [7]. Once bacterial overgrowth is diagnosed, it can be successfully treated using antibiotics (rifaximin, metronidazole, neomycin, chlortetracycline, ciprofloxacin) [8].

Our hypothesis was that some patients diagnosed as suffering exclusively from flatus incontinence might also be suffering from SIBO and might thus be candidates for antibiotic treatments aimed at improving their condition. This hypothesis was based in part on anecdotal case reports in our center of patients reporting a symptomatic improvement of their flatus incontinence following an antibiotic regimen indicated for the treatment of SIBO.

The aims of our pilot randomized trial were to assess the efficacy of an antibiotic (metronidazole) in a select population of patients with flatus incontinence associated with SIBO [9–10] and to compare its efficacy with that of a combination of simethicone and activated charcoal (SC; Carbosylane) (currently used to treat gaseous symptoms) in randomized experimental arms.

## Materials and methods

### Subjects

All patients over 18 years of age who were referred to two specialist motility centers for flatus incontinence between January 2012 and August 2015 performed a glucose breath test. Patients were prospectively enrolled in the study if they met the following inclusion criteria: (i) their glucose breath test was positive for SIBO, (ii) their flatus incontinence was isolated, or predominant in the case of associated fecal incontinence, and (iii) they had suffered from flatus incontinence for at least 3 months prior to being enrolled in the study. Patients were excluded from the study if they (i) had a pre-existing organic disorder, (ii) had taken antibiotics or probiotics within the previous 3 months, (iii) had a history of autonomic diabetic neuropathy or connective tissue disease, (iv) had undergone intestinal surgery (except cholecystectomy or appendectomy), (iv) were pregnant or breast-feeding, and/or (v) were allergic to metronidazole or SC. The study protocol was approved by the Haute-Normandie ethics committee (n°RCB2009-100347-50). The patients provided written informed consent. The study complied with the ethical principles enshrined in the Declaration of Helsinki II and was registered with ClinicalTrials.gov (NCT01275560).

### Design of the trial

The prospective, parallel-group, pilot randomized trial was designed to assess the effect of a 10-day course of metronidazole (1500 mg/day; 500 mg TDS) and to compare the effect with a 10-day course of SC. Each dose of SC consisted of a gastrosoluble capsule containing 45 mg of simethicone and 140 mg of activated charcoal. As recommended in the licensing terms, the patients took three doses per day. Treatments received for conditions other than flatus incontinence were authorized if the treatment did not change during the study period. The patients were also instructed to maintain their usual diets.

At the first visit, after verifying the inclusion criteria and informed consent, the patients were asked to record their symptoms, including the number of gas leakages, on a daily basis in a diary for 3 days prior to the second visit (randomization). At the second visit, the patients were randomly assigned to the 10-day metronidazole or SC treatment using a random number table. The list of random numbers was prepared by the Pharmacy Department of Rouen University Hospital. Members of the Pharmacy Department were not clinically involved in the study. The patients and evaluators were not blinded to the type of treatment. A follow-up evaluation visit was scheduled at the end of the treatment. The patients were asked to complete a bowel diary during the 3 days preceding the follow-up evaluation visit. At the end of the treatment period, the patients who had received metronidazole underwent another glucose breath test to confirm the eradication of SIBO.

### Breath testing

The glucose breath test was performed as previously described [11]. Briefly, 75 g of glucose dissolved in 250 mL of water was administered on the test day, and breath samples were collected at baseline and at 15 min intervals for 2 h in a bag (QuinTron Instrument Company, Inc.). Hydrogen and methane levels in the alveolar gas were analyzed by chromatography (QuinTron Micro Analyzer, QuinTron Instrument Company, Inc.).

The glucose breath test was considered positive for SIBO if at least one of the following criteria was met: (i) ≥10 ppm increase above H_2_ and/or CH_4_ baseline values for at least two consecutive measurements; (ii) ≥10 ppm increase between minimum and maximum H_2_ and/or CH_4_ values over the 2-h breath collection period; and (iii) 20 ppm of H_2_ and/or CH_4_ at baseline if the patient had followed the preparation guidelines^7^.

### Outcome measures

The primary outcome was the percentage decrease in the mean daily number of gas leakages reported in 3-day diaries completed before (baseline period) and at the end of the 10-day treatment period.

Secondary end points included the prevalence of a clinical response defined as a greater than 50% reduction in the mean daily number of gas leakages [12], the frequency of fecal incontinence and urgency episodes, the frequency of bowel movements, symptom scores, and fecal incontinence severity and quality of life scores. Bowel movements and fecal incontinence and urgency episodes were recorded in the diaries completed at baseline and at the end of the treatment. The patients were asked to subjectively rate the intensity of each symptom (bloating, abdominal pain, borborygmi) using the following scores: 0 = none, 1 = mild, 2 = moderate, and 3 = severe. The means of the scores attributed daily for 3 days were then calculated to obtain the final scores for the symptoms at the start and end of the treatment period. Incontinence severity was scored using the Cleveland Clinic Continence Scoring System [13]. Quality of life was assessed using the validated French version of the American Society of Colon and Rectal Surgeons quality of life questionnaire for FI (FIQL) [14] and the gastrointestinal quality of life index (GIQLI) [15]. The fecal incontinence severity and quality of life scores were assessed at baseline and at the end of the treatment. The patients were asked to report all expected and unexpected adverse events during the study. The patients were also queried about adverse events at each scheduled visit during the study. The investigator had to judge whether an event could likely be attributed to the treatments or to the study. All the adverse events attributed to the treatment or to the study are reported herein.

### Data analysis and statistics

Due to the lack of a preliminary study on treatment efficacy for flatus incontinence, we were unable to determine a sample size and decided to arbitrarily enroll 20 patients in each group for the pilot trial.

Values are expressed as medians and ranges or means ± standard deviation. The chi-square test was used to compare qualitative variables. Continuous (numerical) efficacy data and changes from baseline for the two groups were analyzed using the Wilcoxon signed-rank test. Differences in continuous variables between the two groups were compared using the Mann-Whitney U-test. p<0.05 was considered statistically significant for all the analyses. The statistical analyses were performed using Statview version 5.0 with a two-sided significance level of 0.05 and a 95% confidence interval.

## Results

### Patient demographics

**Fig 1:**
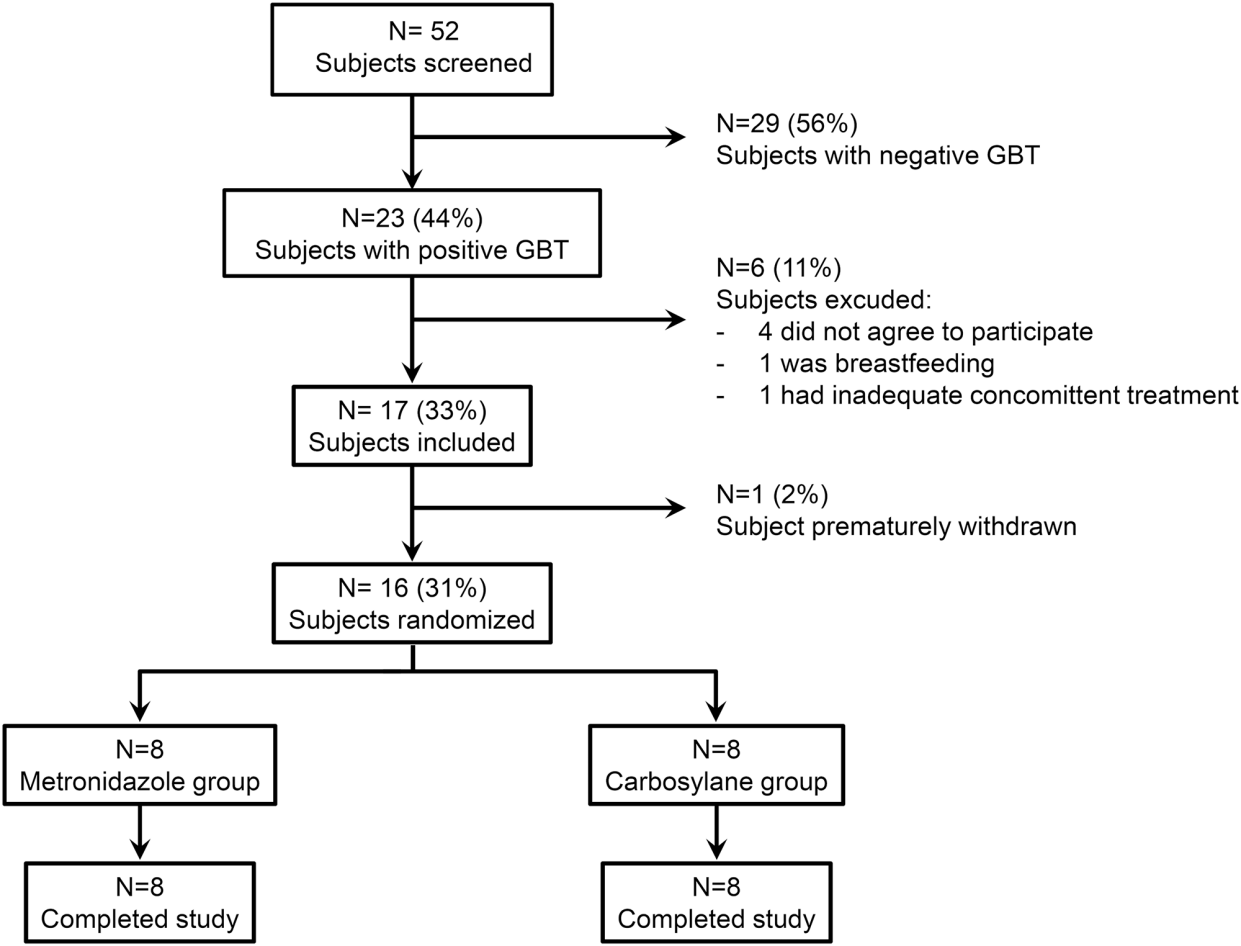
Patient flow chart. Fifty-two consecutive patients were screened from January 2012 to August 2015. Twenty-three (44%) had a positive glucose breath test, indicating SIBO. Seventeen (33%) patients were enrolled in the study. GBT: glucose breath test.

Fifty-two consecutive patients were screened from January 2012 to August 2015 (Fig 1). The patients had been referred to two specialized centers for flatus incontinence and were explored by glucose breath tests to look for possible underlying mechanisms of flatus incontinence. Twenty-three (44%) had a positive glucose breath test, indicating SIBO. Seventeen (33%) patients were enrolled in the study (Fig 1). One patient withdrew before randomization and 16 (31%) (15 females, mean age 61 years [43–79]) completed the study.

Four years after the start of the study, we decided to stop recruiting patients without having reached our goal of 20 patients for each group due to the low recruitment rate of patients who met the inclusion criteria.

The baseline characteristics of the metronidazole and SC groups were similar except for the quality of life scores (Tables 1 and 2). The patients in the metronidazole group had a significantly more altered quality of life than the patients in the SC group (Table 2).

**Table 1:** Comparison of the demographics and baseline glucose breath test results of the metronidazole and SC groups.

Characteristic	Metronidazole N = 8	SC N = 8	P Value
Age (yr.)	60.9±5.9	60.1±11.4	0.83
Gender (male/female)	0/8	1/7	1.0
BMI	27.1±7.3	23±1.9	0.38
Glucose breath test			
H_2_ positive	3	3	0.26
CH_4_ positive	5	4	
H_2_ and CH_4_ positive	0	1	

**Table 2:** Symptoms reported in the bowel diaries; and the Cleveland clinic severity, FIQL, and GIQLI scores of the metronidazole and SC groups at baseline and at the end of the 10-day treatment.

	Baseline			10 days		
	Metronidazole	SC	P	Metronidazole	SC	P
Mean number of gas leakages	18.2±16.2	11.1±12	0.49	3.5±3.1*	8±9.7	0.32
Mean number of fecal incontinence episodes	0.7±1.1	0.2±0.4	0.28	0.2±0.3	0.1±0.3	0.40
Mean number of urgency episodes	0.4±0.6	0.06±0.2	0.12	0.1±0.2	0±0	0.06
Mean number of defecations	2.3±1.2	1.9±0.7	0.53	2.4±2.4	1.3±0.7	0.37
Mean abdominal pain score	1.4±1	1±0.9	0.37	0.9±1.2	0.5±0.8*	0.78
Mean bloating score	1.7±1.1	0.6±0.5	0.06	1±0.8	0.5±0.8	0.19
Mean borborygmi score	0.6±1.1	0.8±0.7	0.26	0.1±1.1	0.7±0.8	0.12
Cleveland Clinic Severity score	9.6±3.7	6.4±3.1	0.07	9.4±3.2	5.6±2.2	0.02
FIQL scores						
Lifestyle	2.8±0.7	3.6±0.4	0.03	2.5±0.9	3.6±0.5	0.05
Coping and behavior	2.2±0.7	3.3±0.5	0.01	2.2±0.6	3.4±0.6	0.007
Depression and self-perception	2.8±0.9	3.8±0.5	0.04	2.8±0.8	3.7±0.7	0.03
Embarrassment	1.9±1	2.7±1	0.1	1.7±0.5	3±0.7	0.002
GIQLI score	84.5±15.2	103.7±17	0.04	90.5±19.7	104.7±17.8	0.19

Mean±standard deviation (SD).

*: P<0.05 versus baseline data in the same group of patients

### Compliance with and effect of the treatments on flatus incontinence

Compliance with the treatment was excellent for the two groups (100%). The glucose breath test results were normal after the metronidazole treatment, indicating that the antibiotic treatment had eradicated the SIBO.

A significant reduction in the mean daily number of gas leakages was observed with metronidazole (P = 0.02) but not with SC (P = 0.09) (Table 2). The relative reduction in flatus incontinence episodes was significantly higher in the metronidazole group than in the SC group (66.8±34.8% vs. 25±50%, respectively, P = 0.03). The number of flatus incontinence episodes decreased by more than 50% in 7 (87.5%) of the patients in the metronidazole group compared with only 1 (12.5%) in the SC group (odds ratio 1.9, 95% confidence interval 0.9–56.9, P = 0.06).

### Effect of the treatments on other digestive symptoms and the severity and quality of life scores

There were no significant differences in the mean scores for abdominal pain, bloating, borborygmi, and the Cleveland Clinic severity and quality of life scores for the patients in the metronidazole group at the end of the treatment period (Table 2). There was a statistically significant reduction in the abdominal pain score (P = 0.04) for the patients in the SC group at the end of the treatment period (Table 2). The significant difference in quality of life observed at baseline between the two groups was still present at the end of the treatment period (Table 2).

### Side-effects

At the 10-day follow-up visit, the self-reported tolerance of the treatment appeared to be better in the SC group than in the metronidazole group. Eight minor adverse events were reported in 4 patients in the metronidazole group: diarrhea (1 case), nausea (1 case), abdominal pain (1 case), metallic taste in the mouth (2 cases), dizziness (1 case), sleepiness (1 case), and urinary frequency (1 case). None of these side-effects led to a treatment interruption. There were no adverse events reported by patients in the SC group.

## Discussion

The results of the pilot controlled phase II study reported here showed that metronidazole given three times a day for 10 days significantly reduces flatus incontinence episodes in patients in whom the incontinence is associated with SIBO. The relative reduction in flatus incontinence episodes was 67% with the metronidazole treatment, which was significantly higher than with the SC treatment (25%). The percentage of improvement for patients in the metronidazole group was close to statistical significance. Several studies have previously shown that antibiotics are more effective than placebos in eradicating SIBO and that the eradication may be correlated with a clinical response^16-18^. However, these studies evaluated diarrhea, abdominal pain or discomfort, bloating, flatulence, nausea, constipation, tenesmus, and weight loss [16–18]. To our knowledge, the present study is the first to suggest that antibiotherapy might be more successful in treating flatus incontinence associated with SIBO than gas absorbents.

The management of flatus incontinence is challenging. Conservative medical (biofeedback) and surgical (sacral nerve stimulation, artificial bowel sphincter) strategies for treating fecal incontinence are often disappointing in reducing gas leakages [19–20]. Exclusion diets appear to be effective in reducing both symptoms and gas production. However, they are often too restrictive to be maintained for long periods [21]. Therapies such as SC have been used to relieve gaseous symptoms, but studies on their efficacy have given contradictory results [9–10]. Our results suggested that antibiotherapy might be a valuable treatment option for patients complaining of flatus incontinence associated with SIBO. However, it must be emphasized that only a small proportion of patients complaining of flatus incontinence can be treated with antibiotics. Of the patients who were referred to tertiary care centers for flatus incontinence and who underwent a breath test, only 44% had an abnormal glucose breath test result pointing to SIBO. While we were unable to estimate the exact prevalence of SIBO in a population of patients suffering from flatus incontinence, the results of our pilot study suggested that SIBO is only involved in flatus incontinence in a minority of patients. Nevertheless, our results indicated that glucose breath tests can be used to detect SIBO and, in the event of a positive result, to prescribe an antibiotic.

The efficacy of metronidazole on other digestive symptoms was disappointing. Fecal incontinence, urgency episodes, and stool frequency were not significantly altered following the antibiotic and SC treatments. Indeed, fecal incontinence severity scores were unchanged in the two treatment groups. This may have been due to the fact that fecal incontinence or urgency episodes and transit disorders were absent, rare, or not predominant in the patients in our study cohort. Unlike a previous study [22], we did observe any significant effect by the metronidazole or SC treatment on bloating or borborygmi. However, the SC treatment significantly decreased the abdominal pain score while metronidazole had no effect. The apparent discrepancy in terms of the efficacy of metronidazole in reducing the excessive involuntary passage of rectal gas but not the feelings of abdominal discomfort, bloating, and borborygmi can be explained by the fact that flatulence is the only complaint that can be attributed to excessive gas production in the colon [23]. The analgesic effect of SC is more difficult to explain. SC is a combination of simethicone, an inert substance with antifoaming activity, and activated charcoal, an absorbent agent. The mechanism of action of simethicone remains unclear. While it is not absorbed, it is supposed to reduce air production in the gastro-intestinal tract and facilitate gastro-intestinal clearance [24]. However, one animal study has shown that simethicone may have a visceral anti-nociceptive effect [25]. This finding has been confirmed in patients with irritable bowel syndrome but only in association with other substances (e.g., alverine citrate, probiotics) [26].

Although the metronidazole treatment significantly reduced flatus incontinence episodes, it did not improve quality of life scores between baseline and follow-up. This may be because, as previously suggested [2], flatus incontinence has less impact upon quality of life than liquid or solid stool loss. However, it is unclear whether this is because the FIQL and GIQLI scores are less sensitive to the changes flatus incontinence causes in a patient’s life or whether flatus incontinence is truly less bothersome. The duration of the follow-up (i.e., 10 days) may also have been too short to have an impact on quality of life [12]. Lastly, it cannot be excluded that the high prevalence of side-effects in the metronidazole group may have a negative impact on quality of life despite the reduction in flatus incontinence episodes.

Our study had several important limitations. It was not double-blinded for two reasons: (1) we did not have the necessary financial support to provide a placebo treatment and (2) we wanted to compare the antibiotic treatment with a treatment routinely used for flatus incontinence. Our patient cohort was also relatively small given the difficulty in finding patients who met the inclusion criteria. However, despite this, the controlled parallel study design was powerful enough to detect a relative reduction in flatus incontinence episodes. Although the percentage of improved patients was higher in the metronidazole group than in the SC group, the difference was not statistically significant. This may have been due to the small number of patients in our study and/or to the fact that the two groups underwent an active treatment (metronidazole or SC) whose efficacy has been already proved and, as such, a clinical improvement was to be expected in the two groups. Another limitation was that, despite the randomization, the two groups were not perfectly balanced. Even if the frequency of flatus incontinence episodes was the same in the two groups, the patients in the metronidazole group appeared to have more severe digestive disorders give their significantly lower quality of life scores. It cannot be excluded that the more severe digestive disorders in the metronidazole group could have influenced the results of our study.

## Conclusion

Our results show a promising trend indicating that metronidazole might significantly improve flatus incontinence associated with SIBO. A multicenter prospective randomized trial with a large patient cohort will be required to definitively prove whether metronidazole is more successful in treating flatus incontinence than gas absorbents. Although further studies are needed to determine the best antibiotic to use and the optimal duration of the treatment, periodic administration of antibiotics might serve as a possible therapeutic approach for the treatment of flatus incontinence associated with SIBO. Lastly, glucose breath tests may be helpful in diagnosing SIBO and monitoring the effectiveness of antibiotherapies in individual patients.

## Supporting information

S1 File Consort 2010 checklist. (DOC)

S2 File Clinical trial protocol (English). (DOCX)

S3 File Clinical trial protocol (French). (DOC)

S4 File Statistics. (DOCX)
