# Supplementary material for: Efficacy of antibiotherapy for treating flatus incontinence associated with small intestinal bacterial overgrowth: A pilot randomized trial
Source: PLoS One. 2017 Aug 1;12(8):e0180835. doi: 10.1371/journal.pone.0180835 (PMC5538639; doi:10.1371/journal.pone.0180835)
Supplement: S2 File — (DOCX) [file pone.0180835.s002.docx]

**Biomedical Research No. RCB 2009-A00347-50**

**Ref. study sponsor: No. 2008/073/HP**

**Version No. 6, February 21, 2010**

**Are the negativation of glucose breath test results associated with a reduction in the symptoms of flatus incontinence?**

**Principal investigator:**

Professor Anne-Marie LEROI

Service de Physiologie Digestive, Urinaire, Respiratoire et Sportive

CHU Charles Nicolle – Hôpitaux de Rouen

76031 ROUEN CEDEX

Telephone: 02-32-88-80-39; Fax: 02-32-88-84-25

Email: anne-marie.leroi@chu-rouen.fr

**Sponsor:**

Direction de la Recherche et de l’Innovation

CHU Rouen

1 rue de Germont

76031 Rouen Cedex

Telephone: 02-32-88-82-65; Fax: 02-32-88-84-25

**Co-investigators:**

**Dr. J. F. QUINTON**

Service Maladies de l’Appareil Digestif et Nutrition

Hôpital Claude Huriez – CHRU Lille

59037 Lille Cedex

**Professor P. DUCROTTE**

Département d’Hépato-Gastroentérologie et Nutrition

CHU Charles Nicolle – Hôpitaux de Rouen

76031 Rouen Cedex

**Dr. G. GOURCEROL**

Service de Physiologie Digestive, Urinaire, Respiratoire et Sportive

CHU Charles Nicolle – Hôpitaux de Rouen

76031 Rouen Cedex

**Statistician:**

Dr. Jean-François Menard

Unité de Biostatistiques

CHU Charles Nicolle – Hôpitaux de Rouen

76031 Rouen Cedex

**SUMMARY**

**1. STATE OF SCIENTIFIC KNOWLEDGE**

**2. AIMS OF THE STUDY**

**3. METHODS**

3.1 Outcome measures

3.2 Inclusion criteria

3.3 Exclusion criteria

3.4 Patient selection

3.5 Breath testing

3.6 Study design and randomization

**4. DATA ANALYSIS AND STATISTICS**

4.1 Number of patients

4.2 Data collection

4.3 Data analysis

**5. STUDY LOCATION**

**6. STUDY DURATION**

**7. MONITORING**

7.1 Serious adverse events

7.2 Unexpected serious adverse events

7.3 Serious adverse events and/or results of analyses used to evaluate patient safety

7.4 Adverse events

7.5 Expected serious adverse events

**8. REGULATORY AND ETHICAL ASPECTS**

8.1 Risk-benefit equation

8.2 Submission to the ethics committee

8.3 Written informed consent of patients

8.4 Information provided to hospital directors

8.5 Declaration of Helsinki, Directive 2001/20/CE

8.6 Insurance

8.7 Right of access to data and document sources

8.8 Confidentiality

8.9 Archiving

**9. PUBLICATIONS**

**10. BIBLIOGRAPHY**

**1. STATE OF SCIENTIFIC KNOWLEDGE**

Anal incontinence is a public health problem because of its prevalence (Denis, 1992). Anal incontinence includes the accidental passing of solid or liquid stools via the rectum (fecal incontinence) and the involuntary leakage of gas (flatus incontinence). Flatus incontinence is the most common type of anal incontinence, with a prevalence estimated at approximately 33% in the general adult population (Damon, 2006). Although flatus incontinence appears to have less impact upon quality of life than fecal incontinence (Boreham, 2005), it is an embarrassing and socially restricting problem (Steinberg, 2009; Meyer, 2015). The management of flatus incontinence is challenging. Conservative medical (biofeedback) and surgical (sacral nerve stimulation, artificial bowel sphincter) strategies used to treat fecal incontinence are often disappointing in terms of reducing gas leakages (Michot, 2003; Leroi, 2009). Medications such as simethicone/activated charcoal are used to relieve gaseous symptoms, but the few studies on their efficacy have reported contradictory results (White, 1991; Friis, 1991).

Flatus incontinence is associated with anal sphincter weakness and can be exacerbated by excessive rectal gas production (Di Stefano, 2000), which can be caused by small intestinal bacterial overgrowth (SIBO). SIBO is defined as a bacterial population in the small intestine exceeding 10^5^–10^6^ organisms/mL (Simren, 2006). Symptoms of SIBO include diarrhea, weight loss, anemia, and intestinal malabsorption. Gastrointestinal tract surgeries that create blind loops (jejuno-ileal bypass, end-to-side enteroenteric anastomosis) as well as small bowel motility disorders (sclerodermia, intestinal pseudo-obstruction) predispose to bacterial stasis and overgrowth. SIBO has also been observed in patients with irritable bowel syndrome or dyspepsia (Pimentel, 2000; Pimentel, 2003). SIBO is generally diagnosed using glucose hydrogen (H_2_) and methane (CH_4_) breath tests.

The principle of the test is as follows: exhaled H_2_ and CH_4_ are produced by the bacterial metabolism of ingested glucose in the colon. Normally, ingested glucose is totally absorbed in the small intestine and does not reach the colon. Absorbed glucose is not metabolized to produce H_2_ or CH_4_. In the case of SIBO, bacteria metabolize glucose before it is absorbed, producing H_2_ or CH_4_ that appears early in breath samples. Since it has a sensitivity of approximately 80%, the glucose breath test is more commonly used to diagnose SIBO than endoscopic small bowel aspirate cultures (Fine, 1999). Although antibiotics can be used to treat SIBO, there is no consensus about the type of antibiotics to use, how often they should be used, or whether a single antibiotic or whether several alternating or combined courses of antibiotics should be prescribed (Quigley, 2006]. However, norfloxacin, amoxicillin-clavulanic acid, and metronidazole appear to be good therapeutic options (Quigley, 2006].

We anticipate that the negativation of glucose breath test results in patients with flatus incontinence, which indicates that SIBO has been successfully eradicated, will result in a symptomatic improvement of their flatus incontinence.

**2. AIMS OF THE STUDY**

The aims of the study are to compare the efficacies of a conventional therapy and of an antibiotic therapy for treating SIBO diagnosed by the glucose breath test and to determine whether the antibiotic treatment improves flatus incontinence.

**3. METHODS**

**3.1 Outcome measures**

The main outcome measure will be the daily number of gas leakages.

The secondary endpoints will be:

- The fecal incontinence severity score
- The quality of life scores
- The frequency of fecal incontinence episodes
- The frequency of urgency episodes
- The frequency of bowel movements
- The symptom scores (abdominal pain, borborygmi)

**3.2 Inclusion criteria**

The inclusion criteria will be:

1. Patients over 18 years of age who were referred to our center for isolated or predominant (in the case of associated fecal incontinence) flatus incontinence
2. Patients who have a positive glucose breath test
3. Patients who have read and signed the informed consent
4. Patients who are affiliated with the national healthcare system
5. Patients who go to the inclusion visit

**3.3 Exclusion criteria**

The exclusion criteria will:

1. Patients who are pregnant or breast feeding
2. Patients who are not undergoing contraceptive treatments
3. Patients who have taken antibiotics and/or probiotics within the previous 3 months
4. Patients who have had a positive glucose breath test in the past
5. Patients with an organic colonic or anorectal disease (cancer, ischemia, infection, etc.)
6. Patients with an autonomic diabetic neuropathy, dysthyroidism, coeliac disease, or connective tissue disease
7. Patients who have undergone intestinal surgery (except cholecystectomy or appendectomy)
8. Patients with a psychiatric disorder
9. Patients who have participated in another medicinal product study during the 30 days preceding the inclusion visit
10. Patients who are allergic to metronidazole
11. Patients who do not speak or read French
12. Patients who are no longer autonomous

Patients who are taking a treatment (laxative, anti-diarrheic treatment, morphine, antidepressant, etc.) that may interfere with their gastro-intestinal motility or sensitivity will be asked to continue their treatment during the study. In the event of a significant change during the study, the patient will be excluded.

**3.4 Patient selection**

All patients who consult for flatus incontinence or for predominant flatus incontinence in the case of associated fecal incontinence (i.e., occasional fecal incontinence episodes with fewer than one episode per month) will be asked if they are interested in participating in the study.

Each patient will complete a self-assessment questionnaire about their continence disorder and their digestive symptoms, including the Rome 3 criteria. Each patient will also undergo a medical examination during which a questionnaire about their medical and surgical histories, current treatments, including contraceptive treatments, will be completed. A clinical examination will also be performed. Patients with anal incontinence will not undergo a comprehensive continence assessment (anorectal manometry, endoanal ultrasound, electrophysiologic tests) because flatus incontinence is often refractory to treatments that are usually indicated for fecal incontinence and because such an assessment will not be helpful in choosing the most appropriate treatment.

**3.5 Breath testing**

Several methods have been used to diagnose SIBO [Romagnuolo, 2002]. We chose the glucose breath test because (1) it provides excellent inter-observer reproducibility (100%), which is not the case, for example, for the lactulose breath test, (2) 100% of a 75 g dose of glucose is completely absorbed in the duodenum and, as such, there is no risk of a positive glucose breath test due to the arrival of glucose in the colon and the transit time in the small intestine does not have to be taken into consideration, and (3) it has the sensitivity and specificity required to diagnose SIBO [Fine, 1999].

The glucose breath test will be performed using a previously published method [Johlin, 2004]. Patients will be asked to avoid ingesting unfermentable carbohydrates (e.g., whole grain breads, pasta) in order to reduce the production of H_2_ during the two days preceding the test. Patients will be asked to fast overnight. Good oral hygiene will be recommended the day before and the morning of the test. Smokers will be asked to avoid smoking for 1–2 h prior to the test. Patients will be asked to avoid exercise for 2 h prior to the test. Patients will be asked to rinse their mouths with a antiseptic chlorhexidine solution prior to the test to eradicate oral bacterial flora. Patients will be asked to ingest 75 g of glucose dissolved in 250 mL of water. Breath samples will be collected at baseline and at 15-min intervals for 2 h in a bag (QuinTron Instrument Company, Inc.). Hydrogen and methane levels in the alveolar gas will be analyzed by chromatography (QuinTron Micro Analyzer, QuinTron Instrument Company, Inc.). Patients will be asked to complete a questionnaire listing their symptoms following the ingestion of the glucose.

The glucose breath test will be considered positive for SIBO if at least one of the following criteria are met: (i) ≥10 ppm increase above H_2_ and/or CH_4_ baseline values for at least two consecutive measurements; (ii) ≥10 ppm increase between minimum and maximum H_2_ and/or CH_4_ values over the 2-h breath collection period; and (iii) 20 ppm of H_2_ and/or CH_4_ at baseline if the patient followed the preparation guidelines [Romagnuolo, 2002].

**3.6 Study design and randomization**

All patients referred for flatus incontinence will undergo a glucose breath test. In the event of a positive glucose breath test, the patients will be asked to complete a 3-day bowel diary prior to randomization.

The patients will receive:

- Metronidazole (Flagyl^®^ per os, 500 mg, 3 per day for 10 days), or
- Carbosylane^®^ (45 mg of simethicone and 140 mg of activated charcoal per capsule, 3 capsules per day for 10 days.

The choice of antibiotic was empirical because of the small number of studies investigating the efficacy of antibiotics for treating SIBO and the lack of consensus. Large spectrum antibiotics appear to be the most appropriate antibiotics since they are effective against both aerobic and anaerobic bacteria. Rifaximin appears to be more effective than tetracycline and is not absorbed after oral ingestion, limiting the secondary effects. Unfortunately, rifaximin is not commercially available in France. One controlled study has shown that metronidazole is more effective than rifaximin in reducing the symptoms of SIBO and returning glucose breath test results to normal values [Di Stefano, 2005]. Another controlled study, which compared norfloxacin, clavulanic acid-amoxicillin, a probiotic, and a placebo [Attar, 1999] showed that norfloxacin and clavulanic acid are more effective for treating diarrhea in patients with SIBO than other treatments. Norfloxacin and metronidazole are the most frequently used antibiotics in France for treating SIBO. The minor and moderate secondary effects of norfloxacin and metronidazole are comparable in frequency. However, norfloxacin causes more frequent serious secondary effects than metronidazole, which is why we opted for metronidazole.

A symptomatic evaluation and a glucose breath test (for patients in the group receiving metronidazole) will be performed after the 10-day treatment.

Patients treated with Carbosylane^®^ may be given a course of antibiotics at the end of the study in the event they have persistent digestive symptoms. The results of this antibiotic treatment will not be taken into consideration for the purposes of the study.

If a patient displays secondary effects or develops an allergy to metronidazole or Carbosylane^®^, he/she will be excluded from the study and will be replaced by another patient. Given the number of patients that will potentially be excluded from the study following their inclusion, we plan on recruiting 50 patients in order to maintain a 20-patient cohort for each experimental arm (Carbosylane^®^ and metronidazole).

The main primary outcome will be the percentage decrease in the mean daily number of gas leakages reported in the 3-day diaries that will be completed before (baseline) and at the end of the 10-day treatment period and before the regimen performed prior to the glucose breath test. A positive clinical response will be defined as a greater than 50% reduction in the mean daily number of gas leakages.

**4. DATA ANALYSIS AND STATISTICS**

**4.1 Number of patients**

Due to the lack of a preliminary study on the efficacy of treatments for flatus incontinence, we were unable to determine a sample size and decided to arbitrarily enroll 20 patients in each group for the pilot study.

To maintain 20 patients in each experimental arm (i.e., 20 patients treated with Carbosylane^®^ and 20 with metronidazole), we plan to recruit 25 patients for each experimental arm to compensate for non-compliant patients and patients who develop allergies or intolerance to treatment, or who are lost at follow-up. The frequency of positive glucose breath tests in patients with flatus incontinence is difficult to estimate because this has never been investigated. However, we presume that one out of three patients will have a positive glucose breath test.

**4.2 Data collection**

**Demographic data, clinical symptoms**: All the data will be collected and will be compiled in individual patient case report forms (CRFs), which will be archived in the investigation center. Information will be collected by the research staff or the investigator from the patient and from his/her medical record.

**Bowel diary**: Three-day bowel diaries describing flatus incontinence episodes (main criterion), bowel movements, urgency episodes, and fecal incontinence episodes will be completed by each patient. The treatment and the secondary effects will be noted in the diaries. The bowel diaries will be completed at the beginning of the study before the treatments and at the end of the treatments.

**Fecal incontinence severity score**: The Cleveland Clinic score will be used (Jorge, 1993). Scores will be recorded at the beginning and at the end of the study.

**Quality of life scores**: Quality of life will be evaluated using two questionnaires that have been validated in French. One is specific for fecal incontinence (FIQL) [Rullier, 2004] while the other is specific for functional digestive disorders (GIQLI) [Slim, 1999]. These questionnaires will be completed at the beginning and at the end of the study.

**Symptom scores**: The patients will be asked to subjectively rate the intensity of each symptom (bloating, abdominal pain, borborygmi) using the following scores: 0 = none, 1 = mild, 2 = moderate, and 3 = severe. The means of the scores attributed daily for 3 days will be calculated to obtain the final scores for the symptoms at the beginning and at the end of the study.

**Treatment compliance**: Treatment compliance will be evaluated by counting the number of tablets remaining at the end of the study.

**4.3 Data analysis**

Results will be expressed as means ± standard deviation. The mean number of flatus incontinence episodes during the 3 days prior to and at the end of the treatment will be calculated. The difference between the mean number of flatus incontinence episodes before and during the treatment and the percentage decrease in the mean daily number of flatus incontinence episodes will be calculated and will compared between the two groups of patients (metronidazole and Carbosylane^®^ groups). The severity scores, quality of life scores, mean number of bowel movements, fecal incontinence episodes, urgency episodes, and symptomatic scores will be compared between the two groups.

**5. STUDY LOCATION**

The study will take place in two centers:

Rouen University Hospital

Lille University Hospital

**6. STUDY DURATION**

Expected first inclusion date: September 2010

Expected last inclusion date: September 2012

Expected end date of the study: October 2012

**7. MONITORING**

**7.1 Serious adverse events**

The following will be considered serious adverse events:

1. Death of the patient
2. Life-threatening event
3. Event resulting in a hospitalization or the prolongation of a hospitalization
4. Event potentially leading to a definitive or long-lasting handicap
5. Any other sort of adverse event deemed serious by the investigator in charge of the declaration of the adverse event

**7.2 Unexpected serious adverse events**

The investigator will immediately notify the sponsor (Direction de la Recherche et de l’Innovation, telephone: 02-32-88-80-39 – fax: 02-32-88-84-25) of any unexpected serious adverse event. The notification will be followed by a detailed written report within 2 days.

All unexpected serious adverse events will be recorded in the patient’s CRF. The investigator will judge whether the event could likely, probably, or unlikely be attributed to the treatment or to the study.

In the event of the death of a patient, the investigator will provide the sponsor with all the complementary information required. This information will also be forwarded to the CPP Nord Ouest I.

An unexpected serious adverse event leading to the death of a patient will be declared to the Health Ministry within 7 days of the date the sponsor was notified of the event. All complementary information will be forwarded to the Health Ministry within 8 days of the event. The sponsor will also forward this information to all the investigators.

All other unexpected serious adverse events will be declared to the Health Ministry within 15 days of the date the sponsor was notified of the event. All complementary information will be forwarded to the Health Ministry within 15 days of the event.

**7.3 Serious adverse events and/or results of analyses used to evaluate patient safety**

The investigator will notify the sponsor (Direction de la Recherche et de l’Innovation, telephone: 02-32-88-80-39 – fax: 02-32-88-84-25) within 2 days of any serious adverse event or any adverse event and/or result of an analysis that is deemed important for the safety of the patient. The notification will be followed by a detailed written report within 2 days. All serious adverse events and all adverse events and/or results of analyses deemed important for the safety of the patient will be recorded in the patient’s CRF. The investigator will judge whether the event could likely, probably, or unlikely be attributed to the treatment or to the study.

**7.4 Adverse events**

The investigator will notify the sponsor (Direction de la Recherche et de l’Innovation, telephone: 02-32-88-80-39 – fax: 02-32-88-84-25) as quickly as possible of any adverse event and will record it in the patient’s CRF. The investigator will judge whether the event could likely, probably, or unlikely be attributed to the treatment or to the study. The sponsor will record all adverse events in a register and will forward copies of the register to the ethics committee (CPP Nord Ouest I) and to AFFSAPS.

**7.5 Expected serious adverse events**

There are no expected serious adverse events related to the glucose breath test. Metronidazole could cause the following serious adverse events: digestive disorders, glossitis, pancreatitis (rare), pruritus, cutaneous eruptions, anaphylactic shock, neuropathy, vertigo, headaches, convulsions, confusion, and hematological disorders. These serious adverse events will be declared using the methodology described in the previous section.

**8. REGULATORY AND ETHICAL ASPECTS**

**8.1 Risk-benefit equation**

Adverse events associated with metronidazole are relatively rare and usually cease following discontinuation of the treatment. Antibiotic treatments may improve flatus incontinence, which can seriously impair a patient’s quality of life.

**8.2 Submission to the ethical committee (CPP)**

This protocol is subject to the opinion of an independent ethics committee (section L 1123-6, French Public Health Code). Authorization must be requested from ANSM (French Competent Authority) (section L 1123-8, French Public Health Code).

Information must be provided to the Directors and Pharmacists of centers participating in the study before the research begins (section L 1123-13, French Public Health Code).

Any substantial amendment to the study on the sponsor’s sole initiative is subject to authorization by the ethics committee and by ANSM (art. L 1123-9).

**8.3 Written consent of patients**

Prior to the conduct of a biomedical research study on a person, the free, informed, and express consent of that person must be collected after they have been informed by the investigator of the objective of the research, the conduct and duration of the study, the benefits, potential risks, and constraints of the trial as well as the nature of the study product and the authorization of the ethics committee (section L. 1122-1, French Public Health Code).

The consent form is to be dated and signed personally by the patient or his/her representative and the investigator (original archived by the investigator, a copy to be given to the patient). Only a doctor-investigator who is registered with the National Order of Physicians and who holds a PhD is authorized by law to obtain the signature for the consent form.

**8.4 Information for hospital directors**

Directors of hospitals participating in the study will be notified of the study before it begins in their hospitals.

**8.5 Declaration of Helsinki**

The study will be conducted in accordance with all legal and regulatory requirements and with the general principles set forth in the International Ethical Guidelines for Biomedical Research Involving Human Subjects (Council for International Organizations of Medical Sciences, 2002), Guidelines for Good Clinical Practice (International Conference on Harmonization, 1996), and the Declaration of Helsinki (World Medical Association, 1996 & 2008).

In addition, the study is to be conducted in accordance with the protocol, the International Conference on Harmonization Guidelines on Good Clinical Practice, and applicable local regulatory requirements and legislation.

In accordance with the French Public Health Code, Act 2004-806 of 09 August 2004 regarding public health policy, Directive 201/020/CE of the European Parliament, and the Council of 04 April 2001 (articles 16, 17, and 18), the “detailed guidance for the request for authorization of a clinical trial on a medicinal product for human use to the competent authorities, notification of substantial amendments and declaration of the end of the trial" European Commission, Brussels ENTR/F2/BL D October 2005.

**8.6 Insurance**

Insurance will be taken out in accordance with French legislation. Patients included in the study will be able to consult the insurance attestation.

**8.7 Right of access to data and documents sources**

During the study, the sponsor or its agent will conduct periodic monitoring visits to ensure that the protocol and GCPs are being followed. The monitors may review source documents to confirm that the data recorded in the patient CRFs are accurate. The investigator and the investigation center are to allow the sponsor, its monitors, or its agents as well as the appropriate regulatory authorities direct access to source documents to perform this verification.

The study site may be subject to review by the independent ethics committee (IEC) and/or to quality assurance audits performed by the sponsor or companies working with or on behalf of the sponsor, and/or to inspection by the appropriate regulatory authorities.

**8.8 Confidentiality**

Results will be anonymized. Co-investigators will not publish any results without the authorization of the principal investigator. The computer processing of the data will be performed according to the MR001 methodology of CNIL.

**8.9 Archiving**

All documentation pertaining to the study (protocol, consent, case report forms, investigator folder, etc.) as well as original documents (laboratory results, X-rays, consultation reports, reports of clinical examinations performed, etc.) must be kept in a secure location and will be considered confidential. The archiving of data will be under the responsibility of the principal investigator and will be performed in accordance with the legislation in force. The principal investigator must keep the data as well as the patient identification list for a minimum period of 15 years after the end of the study.

**9. PUBLICATIONS**

The investigators will agree with the results, the analysis of the results, and the conclusions of the study. The study will be published. The co-authors will be the investigators. The order of the co-authors will be based on the number of patients recruited by each co-author. The first author will be the author who analyzes the data and writes the article.

**10. BIBLIOGRAPHY**

Boreham MK, Richter HE, Kenton KS, Nager CW, Gregory T, Aronson MP, Vogt VY, McIntire DD, Schaffer JI. Anal incontinence in women presenting for gynecologic care: prevalence, risk factors, and impact upon quality of life. Am J Obstet Gynecol 2005; 192: 1637-42.

Damon H, Guye O, Seigneurin A, et al. [Prevalence of anal incontinence in adults and impact on quality-of-life](http://www.ncbi.nlm.nih.gov/pubmed/16514381). Gastroenterol Clin Biol 2006; 30: 37-43.

Denis, 1992

Di Stefano M, Strocchi A, Malservisi S, Veneto G, Ferrieri A, Corazza R. Non-absorbable antibiotics for managing intestinal gas production and gas-related symptoms. Aliment Pharmacol Ther 2000; 14: 1001-1008.

Friis H, Bodé S, Rumessen JJ, Gudmand-Hoyer E. Effect of simethicone on lactulose-induced H2 production and gastrointestinal symptoms. Digestion 1991; 49: 227-30.

Johlin FC, Panther M, Kraft N. Dietary fructose intolerance: diet modification can impact self-rated health and symptom control. Nutr Clin Care 2004; 7: 92-7.

Jorge JM, Wexner SD. Etiology and management of fecal incontinence. Dis Colon Rectum 1993; 36: 77 – 97.

Leroi AM, Damon H, Faucheron JL, Lehur PA, Siproudhis L, Slim K, Barbieux JP, Barth X, Borie F, Bresler L, Desfourneaux V, Goudet P, Huten N, Lebreton G, Mathieu P, Meurette G, Mathonnet M, Mion F, Orsoni P, Parc Y, Portier G, Rullier E, Sielezneff I, Zerbib F, Michot F. Sacral nerve stimulation in faecal incontinence: position statement based on a collective experience. Colorect Dis 2009; 11: 572-583.

Meyer I, Tang Y, Szychowski JM, Richter HE. The differential impact of flatal incontinence in women with anal versus fecal incontinence. Female Pelvic Med Reconstruct Surg 2015; 21: 339-42.

Michot F, Costaglioli B, Leroi AM, Denis P. Artificial anal sphincter in severe fecal incontinence. Ann Surg 2002; 237: 52-56.

Nelson RL. Epidemiology of fecal incontinence. Gastroenterology 2004; 126: S3-7.

Romagnuolo J, Schiller D, Bailey RJ. Using breath tests wisely in a gastroenterology practice: an evidence-based review of indications and pitfalls in interpretation. Am J Gastroenterol 2002; 97: 1113-1126.

Rullier EZF, Marel A, Amouretti M, Lehur PA. Validation of the French version of the fecal incontinence quality of life scale (FIQL). Gastroenterol Clin Biol 2004; 28: 562-8.

Simren 2006

Steinberg AC, Collins SA, O’Sullivan DM. The impact of flatal incontinence on quality of life. Am J Obstet Gyn 2009; 201: 539e1-3.

White JG, Hightower NC, Riggs M, Dyck WP. Does activated charcoal relieve gas symptoms? - a placebo controlled study. Gastroenterology 1991; 100: A261 (Abstract).
