# Supplementary material for: Efficacy of antibiotherapy for treating flatus incontinence associated with small intestinal bacterial overgrowth: A pilot randomized trial
Source: PLoS One. 2017 Aug 1;12(8):e0180835. doi: 10.1371/journal.pone.0180835 (PMC5538639; doi:10.1371/journal.pone.0180835)
Supplement: S3 File — (DOC) [file pone.0180835.s003.doc]

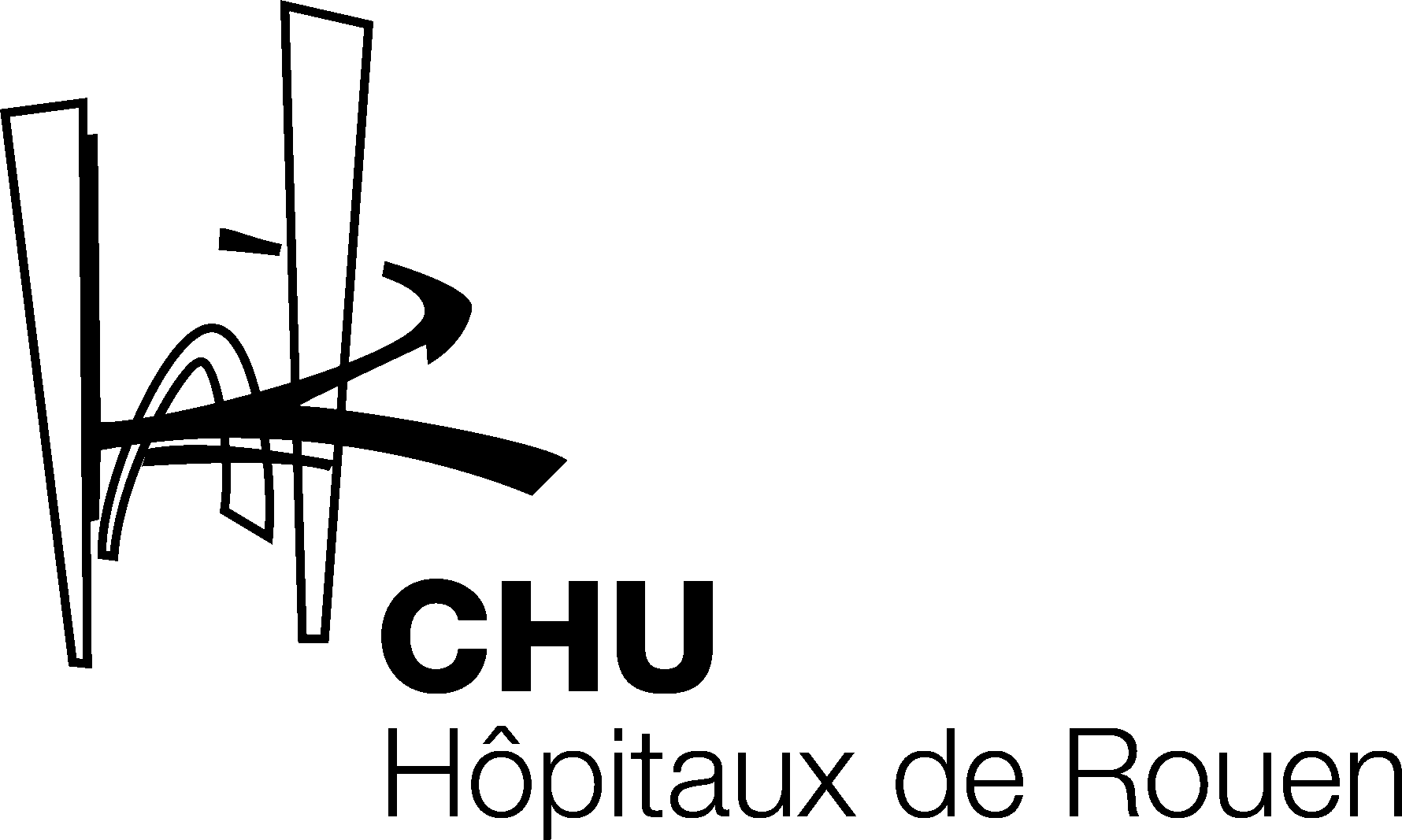


Direction de la Recherche et de l'Innovation

RECHERCHE BIOMEDICALE n° RCB 2009-A00347-50

réf. Promoteur : n°2008/073/HP

Version n°5 du 20/04/09

*PHRC InterRégional 2008*

**La négativation du test au glucose s’accompagne-t-elle d’une réduction des symptômes d’incontinence anale aux gaz ?**

**Investigateur Coordonnateur :**

Pr. Anne-Marie LEROI

Service de Physiologie Digestive, Urinaire, Respiratoire et Sportive

CHU Charles Nicolle – Hôpitaux de Rouen

76031 ROUEN CEDEX

🕿 02 32 88 87 85 🖨 02 32 88 84 25

@ anne-marie.leroi@chu-rouen.fr

**Promoteur :**

Direction de la Recherche et de l'Innovation

CHU de Rouen

1 rue de Germont

76031 Rouen Cedex

Tél. : 02 32 88 82 65

Fax : 02 32 88 82 87

#### Coordonnées des Investigateurs

Co-investigateurs

Dr F Guillemot

Service Maladies de l’Appareil Digestif et Nutrition

Hôpital Claude Huriez - CHRU de Lille

59037 Lille Cedex.

Dr JF Quinton

Service Maladies de l’Appareil Digestif et Nutrition

Hôpital Claude Huriez – CHRU de Lille

59037 Lille Cedex.

Pr Philippe Ducrotté

Département Hépato-Gastroentérologie et Nutrition

CHU Charles Nicolle – Hôpitaux de Rouen

76031 ROUEN CEDEX

Dr Guillaume Gourcerol

Service de Physiologie Digestive, Urinaire, Respiratoire et Sportive

CHU Charles Nicolle – Hôpitaux de Rouen

76031 ROUEN CEDEX

Expert statisticien

Dr Jean-François Menard

Unité de Biostatistiques

CHU Charles Nicolle – Hôpitaux de Rouen

76031 ROUEN CEDEX

SOMMAIRE

[1. Etat des connaissances scientifiques 8](#__RefHeading___Toc228010547)

[2. Objectif de l'étude 9](#__RefHeading___Toc228010548)

[3. Méthodes 9](#__RefHeading___Toc228010549)

[3.1. Critère d’évaluation 9](#__RefHeading___Toc228010550)

[3.2. Sujets incontinents anaux 9](#__RefHeading___Toc228010551)

[3.2.1. Critères d’inclusion 9](#__RefHeading___Toc228010552)

[3.2.2. Critères de non-inclusion 10](#__RefHeading___Toc228010553)

[3.3. Sujets volontaires sains 10](#__RefHeading___Toc228010554)

[3.3.1. Critères d’inclusion 10](#__RefHeading___Toc228010555)

[3.3.2. Critères de non-inclusion 11](#__RefHeading___Toc228010556)

[3.4. Sélection des sujets 11](#__RefHeading___Toc228010557)

[3.5. Recherche d’une pullulation microbienne par l’analyse des gaz expirés après ingestion de glucose (test au glucose) 12](#__RefHeading___Toc228010558)

[3.6. Déroulement de l'essai et randomisation 12](#__RefHeading___Toc228010559)

[4. Analyses statistiques 14](#__RefHeading___Toc228010560)

[4.1. Nombre de sujets 14](#__RefHeading___Toc228010561)

[4.2. Recueil des données 14](#__RefHeading___Toc228010562)

[4.3. Analyse des données 15](#__RefHeading___Toc228010563)

[5. Lieux de l'étude 15](#__RefHeading___Toc228010564)

[6. Durée présumée de l'étude 16](#__RefHeading___Toc228010565)

[7. Vigilance 16](#__RefHeading___Toc228010566)

[7.1. Evénements Indésirables Graves (EIG) 16](#__RefHeading___Toc228010567)

[7.2. Evénements Indésirables Graves Inattendus 16](#__RefHeading___Toc228010568)

[7.3. Evénements Indésirables Graves et/ou résultat d'analyse déterminant pour l'évaluation de la sécurité des patients 16](#__RefHeading___Toc228010569)

[7.4. Evénements indésirables 17](#__RefHeading___Toc228010570)

[7.5. Evénements indésirables graves attendus 17](#__RefHeading___Toc228010571)

[8. ASPECTS REGLEMENTAIRES ET ETHIQUES 17](#__RefHeading___Toc228010572)

[8.1. Balance Bénéfice-Risque 17](#__RefHeading___Toc228010573)

[8.2. Soumission au Comité de Protection des Personnes (C.P.P) 17](#__RefHeading___Toc228010574)

[8.3. Consentement écrit des patients et des volontaires sains 18](#__RefHeading___Toc228010575)

[8.4. Information aux directeurs hospitaliers 18](#__RefHeading___Toc228010576)

[8.5. Déclaration d'Helsinki, Directive 2001/20/CE 18](#__RefHeading___Toc228010577)

[8.6. Assurance 18](#__RefHeading___Toc228010578)

[8.7. Droit d'accès aux données et aux documents source 18](#__RefHeading___Toc228010579)

[8.8. Confidentialité 18](#__RefHeading___Toc228010580)

[8.9. Archivage 19](#__RefHeading___Toc228010581)

[9. Publications 19](#__RefHeading___Toc228010582)

[10. Références bibliographiques 20](#__RefHeading___Toc228010583)

[11. ANNEXES 22](#__RefHeading___Toc228010584)

# Etat des connaissances scientifiques

Les troubles de la continence anale (incontinence anale) et, parmi eux, l’incontinence aux gaz, constituent, de par leur fréquence, un problème de santé publique [Denis, 1992]. L’incontinence aux gaz est définie par l’impossibilité de différer volontairement l’émission de gaz**.** Une enquête postale réalisée auprès de 2800 personnes âgées de plus de 18 ans, domiciliées dans la région Rhône Alpes, a montré que 39% des personnes interrogées présentaient une incontinence aux gaz [Damon, 2006]. L’incontinence aux gaz peut être très invalidante, dégradant significativement la qualité de vie des patients. Pourtant, peu de solutions thérapeutiques existent pour ce type d’incontinence. Les traitements médicaux (traitements laxatifs, diarrhéiques), la rééducation périnéale ainsi que les traitements chirurgicaux (sphinctérorraphie, stimulation des racines sacrées, sphincter anal artificiel) dont nous disposons pour améliorer la compétence sphinctérienne, sont souvent inefficaces pour l’incontinence aux gaz [Michot, 2003 ; Leroi, 2009]. De la même façon, l’utilisation de traitements absorbant les gaz (Carbosylane ®) est souvent décevante [white, 1991; Friis, 1991].

L’incontinence aux gaz peut être favorisée par une incompétence sphinctérienne mais également par une production intestinale excessive de gaz [Di Stefano, 2000]. La production excessive de gaz peut se rencontrer en cas de pullulation microbienne intestinale. La pullulation microbienne intestinale est définie par la présence d’une quantité anormale de bactéries normalement présentes dans la lumière intestinale (i.e >10^5^ unités formant une colonie de bactéries/mL de liquide jéjunal) [Simren, 2006]. Elle se traduit par une diarrhée, une perte de poids, une anémie, et une malabsorption intestinale. Elle est classiquement décrite chez des patients présentant un facteur anatomique (anse borgne, sténose partielle, diverticules du grêle) ou fonctionnel (sclérodermie, pseudo-obstruction intestinale chronique du grêle, achlorhydrie gastrique) favorisant. Plus récemment, elle a également été observée chez des patients souffrant de colopathie fonctionnelle et/ou de dyspepsie [Pimentel, 2000; Pimentel, 2003]. Son diagnostic repose sur le test respiratoire à l’hydrogène (H_2_) et au méthane (CH_4_) après ingestion de glucose (Figure 1). Le principe du test est le suivant : l’H_2_ et le CH_4_ de l’air expiré ne proviennent que de la fermentation colique des glucides. Normalement absorbé en totalité par l’intestin grêle, le glucose ne parvient pas au côlon ; il n’induit donc pas d’expiration d’H_2_ ou de CH_4_. En cas de pullulation bactérienne intra-luminale du grêle, les bactéries métabolisent le glucose avant son absorption et produisent rapidement de l’H_2_ ou du CH_4_ qui apparaissent précocement dans l’air expiré. Ce test a, au cours des pullulations chroniques du grêle, une sensibilité et une spécificité diagnostiques de l’ordre de 80%, ce qui a conduit des experts internationaux à le proposer en alternative aux tests de référence que sont les prélèvements bactériologiques intra-luminaux [Fine, 1999]. Lorsqu’il existe une pullulation microbienne intestinale, une antibiothérapie permettra, non pas d’éradiquer la flore intestinale, mais plutôt de la modifier de façon à obtenir une amélioration de la symptomatologie digestive. Il n’existe pas de consensus quant à la classe d’antibiotique à prescrire, la fréquence des cures, l’utilisation d’une monothérapie ou de plusieurs antibiotiques utilisés successivement ou dans le même temps [Quigley, 2006]. La norfloxacine, l’amoxicilline-acide clavulanique et le metronidazole apparaissent être de bonnes options thérapeutiques [Quigley, 2006].

Nous espérons par ce travail montrer qu’un test au glucose, en faveur d’une pullulation microbienne intestinale, est plus fréquemment positif chez les patients souffrant d’incontinence aux gaz comparé à des sujets volontaires sains.

# Objectif de l'étude

Notre hypothèse étant que l’incontinence aux gaz pourrait être favorisée par une production anormale de gaz secondaire à une pullulation microbienne. Les objectifs de ce travail sont les suivants :

**~~Objectif primaire~~**

- Evaluer l’efficacité à court terme du traitement antibiotique en cas d'incontinence au gaz due à une pullulation microbienne en le comparant au traitement habituellement prescrit.

**~~Objectif secondaire~~**

- **~~Comparer la fréquence du test au glucose positif chez des patients souffrants d'incontinence aux gaz et des sujets volontaires sains.~~**

# Méthodes

## Critère d’évaluation

**Le critère d’évaluation principal sera :**

**1/ le nombre quotidien d’accidents d’incontinence aux gaz;**

**Les critères d’évaluation secondaires seront :**

1/ le score de gravité de l’incontinence anale

2/ les scores de qualité de vie

3/ le nombre d’accidents d’incontinence aux selles et impériosités

4/ le nombre de selles par semaine

5/ les scores symptomatiques (douleurs, ballonnements etc..)

## Sujets incontinents anaux

## Critères d’inclusion

1. Patients âgés d’au moins 18 ans, consultant pour une incontinence aux gaz (i.e incapacité de retenir les gaz si on le souhaite), ou présentant une incontinence aux gaz prédominante (i.e, avec une incontinence pour les selles très occasionnelle) datant d’au moins 3 mois
2. Patients ayant un test au glucose positif
3. Ayant lu la lettre d’information et ayant signé le consentement éclairé (Annexes 1 et 2)
4. Affiliés au régime de la Sécurité Sociale
5. Ayant effectué la visite d’inclusion

## Critères de non-inclusion

1. Femmes enceintes ou n’ayant pas de moyen de contraception efficace et en mesure de procréer ou allaitant (en raison de la prescription d’une antibiothérapie en cas de pullulation microbienne intestinale)
2. Patients ayant pris des antibiotiques, des probiotiques, depuis moins de 3 mois (car cela risque de fausser la recherche de pullulation microbienne)
3. Patients ayant déjà effectué des tests respiratoires pour recherche de pullulation microbienne
4. Patients ayant une pathologie colique ou anorectale inflammatoire, ischémique, cancéreuse, infectieuse, devant faire l’objet d’une prise en charge spécifique
5. Patients ayant des antécédents de diabète avec atteinte du système nerveux autonome, de dysthyroïdie non équilibrée, de connectivite, de maladie coeliaque
6. Patients allergiques ou présentant une contre-indication au Metronidazole
7. Patients ayant une pathologie psychiatrique
8. Patients ayant participé à un essai clinique dans les 30 jours précédant la visite d’inclusion ;
9. Patients comprenant mal le français parlé ou écrit ;
10. Patients sous tutelle ou curatelle.

Pour les patients prenant un traitement susceptible de modifier la motricité et/ou la sensibilité colique et/ou anorectale (laxatifs, traitements anti-diarrhéiques, morphiniques, anti-dépresseurs, …), il leur sera demandé de ne pas modifier ce traitement au cours de l’étude sans en avertir les investigateurs. En cas de modification significative (arrêt ou introduction d’un traitement, changements significatifs de doses…), le patient sera exclu de l’étude.

## Sélection des sujets

La participation à cette étude sera proposée à chaque patient consultant pour incontinence aux gaz ou présentant une incontinence aux gaz prédominante (i.e, avec une incontinence pour les selles très occasionnelle, c'est-à-dire inférieure à une fois par mois).

Chaque patient remplira un auto-questionnaire concernant les troubles de la continence anale, les principaux symptômes digestifs en particulier ceux correspondant aux critères de Rome 3. Chaque sujet et patient aura une visite médicale comprenant un interrogatoire concernant ses antécédents médicaux et chirurgicaux, les traitements et moyens de contraception (si nécessaire) utilisés par le sujet. Un examen clinique, en particulier abdominal, sera effectué dans le centre investigateur. Les patients incontinents anaux n’effectueront pas forcément de bilan exhaustif de leur incontinence anale (manométrie anorectale, échographie, tests électrophysiologiques..) car, l’incontinence aux gaz étant résistante aux traitements habituellement proposés en cas d’incontinence, ce bilan n’orientera pas la prise en charge du patient.

## Recherche d’une pullulation microbienne par l’analyse des gaz expirés après ingestion de glucose (test au glucose)

Plusieurs méthodes ont été utilisées pour mettre en évidence une pullulation microbienne [Romagnuolo, 2002]. Nous avons choisi le test au glucose pour plusieurs raisons : 1/ l’interprétation du test est reproductible entre deux observateurs avec une concordance évaluée à 100%, ce qui n’est pas le cas par exemple du test au lactose ; 2/ 100% d’une charge en glucose inférieure à 80g est absorbée par l’intestin grêle. Il n’y a donc aucun risque qu’une augmentation des gaz expirés soit due à l’arrivée de glucose dans le colon. On élimine ainsi la nécessité de devoir tenir compte de la vitesse du transit de l’intestin grêle pour l’interprétation des résultats ; 3/ le test au glucose a une sensibilité et une spécificité correcte pour la recherche de pullulation microbienne [Fine, 1999].

Le test au glucose sera effectué selon une méthodologie validée et précédemment décrite [Johlin, 2004]. Afin de réduire la production d’H_2_ avant le test, le patient fera un régime pendant les deux jours précédant le test excluant les aliments riches en fibres alimentaires, sources de gaz intestinaux pouvant fausser l’examen (le régime est donné en annexe 6). Le patient sera à jeun depuis au moins 12 heures le jour de l’examen. Une bonne hygiène orale est préconisée la veille et le matin de l’examen. Le patient ne doit pas fumer, ni faire d’exercice physique susceptible d’induire une hyperventilation dans les 2 heures qui précèdent le test. Enfin, avant l’examen, le patient fera un lavage de bouche avec une solution antiseptique afin d’éliminer la flore bactérienne orale (chlorhexidine). Les patients ingèreront 75g de glucose dilué dans 250 mL d’eau stérile après avoir fait un recueil basal des gaz expirés. Le recueil des gaz expirés sera effectué toutes les 15 minutes pendant les 2 heures qui suivront la prise orale de glucose à l’aide d’un double sac de recueil. Ce double sac comprend une pièce buccale, une tubulure et un sac de premier recueil pour l’air de l’espace mort, et un sac de recueil terminal pour l’air alvéolaire. C’est à partir de ce dernier sac que seront dosés les gaz expirés. Le dosage du taux d’H_2_ et de CH_4_ sera effectué à l’aide d’un appareil Quintron Microlyzer (Modèle DP, Quintron instrument) et exprimés en particules par million (ppm). Le patient devra noter sur une feuille d’évènements, tous les symptômes ressentis après ingestion du glucose.

Le diagnostic de pullulation microbienne sera effectué si au moins un des critères suivants est rempli : 1) une augmentation d’au moins 10 ppm d’H_2_ et/ou CH_4_ au dessus de la ligne de base sur deux mesures consécutives dans le période de 2 heures ; 2) et/ou une augmentation d’au moins 10 ppm d’H_2_ et/ou CH_4_ entre la valeur minimale et maximale après ingestion du glucose ; 3) et/ou un taux d’H_2_ et/ou CH_4_ supérieur à 20 ppm avant l’ingestion du glucose à condition que le patient ait strictement suivi le régime préconisé avant l’examen [Romagnuolo, 2002].

## Déroulement de l'essai et randomisation

Il sera proposé aux patients consultants pour une incontinence aux gaz **et ayant un test au glucose positif de participer à cette étude. Après le test au glucose, il leur sera demandé d'évaluer quotidiennement leur incontinence aux gaz pendant 3 jours sans traitement puis ils seront traités** :

- soit par un traitement antibiotique (Flagyl®) **~~et un régime alimentaire sans résidu strict~~** pendant 10 jours
- soit par un traitement par Carbosylane® **~~et un régime alimentaire sans résidu strict~~** pendant 10 jours. Le traitement par Carbosylane® (charbon activé) a été choisi car il s’agit du traitement médicamenteux usuel pour tenter de diminuer la production de gaz.

**Le traitement sera déterminé par randomisation.**

Le choix des antibiotiques utilisés pour le traitement de la pullulation microbienne reste empirique compte-tenu du faible nombre d’études contrôlées existantes. Les antibiotiques choisis sont ceux qui ont un large spectre d’action (bactéries aérobies et anaérobies). Parmi ces antibiotiques, la Rifaximine semble plus efficace que les tétracyclines et a l’avantage d’être peu absorbé au niveau intestinal ce qui en limite les effets secondaires (Di Stephano, 2000). Le problème est que la Rifaximine n’est pas commercialisée en France. Par ailleurs, une étude contrôlée a montré une plus grande efficacité à la fois sur les symptômes et sur la négativité du test au glucose du Metronidazole (Flagyl®) versus Rifaximine (Di Stephano, 2005). Une seule étude contrôlée a comparé l’effet d’un placebo, de la Norfloxacine, d’Amoxiciline-Acide clavulanique et probiotique (Attar, 1999). La Norfoxacine et l’Amoxicilline-Acide clavulanique ont démontré une amélioration significative de la diarrhée chez les patients ayant une pullulation intestinale. Au vue de ces études, les antibiotiques les plus utilisées en France pour le traitement de la pullulation microbienne intestinale sont la Norfloxacine et le Metronidazole. Les effets secondaires les plus fréquents de la Norfloxacine et du Metronidazole sont comparables en gravité et en fréquence (notamment effets digestifs et neurologiques). Par contre, les effets peu fréquents (<0.1%) mais sévères de la Norfloxacine sont plus nombreux que ceux du Metronidazole (cf tableau donné en annexe). Pour cette raison, nous décidons de privilégier le Métronidazole.

Une évaluation symptomatique et un test au glucose seront de nouveau effectués à la fin de ces deux périodes de traitement de 10 jours.

Les patients traités par Carbosylane® pourront, en cas de persistance de leurs symptômes et de positivité du test au glucose, effectuer un traitement antibiotique dans un second temps. Chez ces patients, les résultats sur l’incontinence aux gaz après cette période de traitement antibiotique ne seront pas pris en compte compte-tenu du biais potentiel induit par la prise préalable de Carbozylane®. Le Flagyl® sera dispensé à la dose de 500mg trois fois par jour, et le Carbosylane®, 3 prises par jour pendant 10 jours. En cas d’intolérance ou d’inefficacité au Flagyl®, le patient sera exclu de l’étude. Il sera alors remplacé par un autre patient ayant un test au glucose positif, de sévérité équivalente au patient exclu. Comme il est prévisible que plusieurs patients seront exclus de l’étude en raison d’une intolérance/allergie et/ou résistance au Flagyl®, nous inclurons 50 patients de façon à garder 20 patients par bras (i.e; Carbosylane® et régime alimentaire versus antibiothérapie).

Le critère principal d’évaluation principal **sera le pourcentage de diminution du nombre moyen d’accidents d’incontinence aux gaz relevés quotidiennement pendant 3 jours avant le traitement puis à la fin de la période de traitement de 10 jours et en dehors du régime nécessaire pour la réalisation du test respiratoire au glucose. Une réponse clinqiue positive sera définie par une réduction d’au moins 50% du nombre moyen d’accidents d’incontinence aux gaz.**

# Analyses statistiques

## Nombre de sujets

Dans la mesure où cette étude est innovante, nous ne disposons pas de résultats préliminaires nous permettant de faire un calcul d’effectif. Nous proposons de réaliser une étude préliminaire en incluant 40 patients avec un test positif.

Afin d’obtenir 20 sujets dans chaque groupe de patients (i.e patients traités par Carbosylane® **~~et régime alimentaire~~** versus patients traités par Flagyl® **~~et régime alimentaire~~**), nous prévoyons d’inclure 25 sujets par groupe afin de tenir compte des patients non compliants aux traitements, intolérants ou résistants aux antibiotiques, ainsi que des éventuels patients perdus de vue. La fréquence de la pullulation microbienne chez les patients incontinents aux gaz est difficile à évaluer et n'a jamais été étudiée. On peut cependant estimer qu'un patient sur 3 sera positif à l'issue du test respiratoire au glucose.

## Recueil des données

- ***Données socio-démographiques, cliniques* :** l’ensemble des données relatives à chaque patient et volontaire sain sera consigné dans un cahier d’observation standardisé gardé dans le service recruteur. Ces informations seront recueillies par un TEC recruté localement sous la responsabilité du médecin investigateur, ou le médecin investigateur auprès du patient et/ou par consultation des dossiers médicaux.
- ***Calendrier des selles* :** un calendrier sera réalisé, notant la fréquence des accidents d’incontinence aux gaz (**critère principal**), la fréquence des selles et le nombre éventuel de besoins impérieux et d’accidents pour les selles liquides ou solides. Les symptômes tels que les douleurs abdominales, les ballonnements, les borborygmes seront cotés de 0 à 3 selon leur intensité (0 étant équivalent à une absence de symptôme, 1 à une intensité mineure, 2 à une intensité modérée, et 3 à une intensité sévère) (Annexe 7). Les médicaments pris et les éventuels effets secondaires seront également notés. Le calendrier sera effectué au début de l’étude pendant 3 jours et pendant les 3 jours qui précèdent la fin de chaque période.
- ***Score de sévérité de l’incontinence anale :*** Le score choisi est celui de la Cleveland Clinic (Jorge, 1993). Il sera effectué au début de l’étude et à la fin de chaque période de l’étude.
- ***Evaluation de la qualité de vie*** *:* la qualité de vie sera évaluée par deux scores validés en français, l’un spécifique des troubles de la continence anale (FIQL) [Rullier, 2004], le second spécifique des troubles digestifs fonctionnels (GIQLI) [Slim, 1999]. Ces questionnaires seront effectués au début de l’étude et à la fin de chaque période.
- ***Compliance aux traitements ~~et régime~~*** : la compliance au traitement antibiotique sera évaluée en comptant le nombre de comprimés restant à la fin du traitement. **~~La compliance au régime alimentaire sera évaluée par un auto-questionnaire qui sera croisé avec un interrogatoire du patient par le médecin [Sheperd, 2006]. En fonction de ces résultats, le patient sera classé en non-adhérent/adhérent et parmi ces deux classes, une sous-classification (jamais/occasionnellement/fréquemment/toujours) sera réalisée.~~**

## Analyse des données

Les résultats seront donnés sous la forme de moyennes + erreur standard. **Le nombre moyen d’accidents d’incontinence aux gaz durant les 3 derniers jours de chaque période sera calculé. La différence entre le nombre moyen d’accidents d’incontinence aux gaz initial et final ainsi que les pourcentages de diminution des accidents d’incontinence aux gaz seront calculés et comparés entre les deux groupes de patients (i.e; Métronidazole et Carbosylane®).** L**es scores de gravité de l’incontinence anale, les scores de qualité de vie, le nombre moyen d’accidents d’incontinence aux selles, de besoins impérieux, le nombre de selles par semaine, les scores symptomatiques entre les deux périodes, seront comparés entre les périodes initiales et finales et entre les deux groupes**.

# Lieux de l'étude

L'étude se déroulera dans 2 centres.

1. CHU de Rouen
2. CHRU de Lille

# Durée présumée de l'étude

**Date de première inclusion : Mars 2012**

**Date de fin d'inclusion (présumée) : Mars 2015**

**Dernier patient – dernière visite : Avril 2015**

# Vigilance

## Evénements Indésirables Graves (EIG)

Sont considérés comme événements indésirables graves :

- un décès du patient
- un événement mettant en jeu la vie du patient
- un événement indésirable nécessitant une hospitalisation ou une prolongation de son hospitalisation initiale
- un événement risquant d'entraîner un handicap définitif ou durable
- tout autre événement indésirable jugé grave par l'investigateur déclarant l'événement

## Evénements Indésirables Graves Inattendus

L'investigateur notifiera immédiatement au promoteur (la Direction de la Recherche et de l'Innovation, représentant du promoteur (tel : 02 32 88 82 65 – fax : 02 32 88 82 87)) tous les événements indésirables graves inattendus. Cette notification immédiate sera suivie sous un délai maximum de 48h d'un rapport écrit détaillé .

Tout évènement indésirable inattendu survenu pendant la période de l'essai sera documenté sur le cahier de protocole. L'investigateur jugera si l'événement indésirable grave inattendu pourrait être rapporté de façon certaine, probable ou peu probable au traitement administré ou au protocole.

En cas de décès, l'investigateur communiquera au promoteur tous les renseignements complémentaires demandés. Ceux ci seront transmis par le promoteur au CPP Nord Ouest I.

Toute suspicion d'évènement indésirable inattendu ayant entraîné la mort ou pouvant entraîner la mort du patient sera déclarée au Ministère de la santé sous 7 jours calendaires à compter du moment où le promoteur aura pris connaissance des informations s'y reportant et sous 8 jours calendaires pour toute information complémentaire. Ces informations seront également transmises par le promoteur aux investigateurs associés de l'essai.

Toute autre suspicion d'événement indésirable inattendu sera déclaré au Ministère de la santé sous 15 jours calendaires à compter du moment où le promoteur aura pris connaissance des informations s'y reportant et sous 15 jours calendaires pour toute information complémentaire.

## Evénements Indésirables Graves et/ou résultat d'analyse déterminant pour l'évaluation de la sécurité des patients

L'investigateur notifiera dans un délai de 48h au promoteur (la Direction de la Recherche et de l'Innovation, représentant du promoteur (tel : 02 32 88 82 65 – fax : 02 32 88 82 87)) tous les événements indésirables graves ou tout événement indésirable et/ou résultat d'analyse déterminant pour l'évaluation de la sécurité des patients. Cette notification sous un délai maximum de 48h se fera sous la forme d'un rapport écrit détaillé. Tout événement indésirable grave et/ou résultat d'analyse déterminant pour l'évaluation de la sécurité des patients survenu pendant la période de l'essai sera documenté sur le cahier de protocole. L'investigateur jugera si l'événement pourrait être rapporté de façon certaine, probable ou peu probable au traitement administré ou au protocole.

## Evénements indésirables

L'investigateur notifiera le plus rapidement possible au promoteur (la Direction de la Recherche et de l'Innovation, représentant du promoteur (tel : 02 32 88 82 65 – fax : 02 32 88 82 87)) tous les événements indésirables survenus pendant la période de l'essai et les documentera sur le cahier de protocole. L'investigateur jugera si l'événement pourrait être rapporté de façon certaine, probable ou peu probable au traitement administré ou au protocole. Le promoteur tiendra un registre détaillé de ces événements qui seront transmis tous les ans au CPP Nord Ouest I ainsi qu'à l'AFFSAPS.

## Evénements indésirables graves attendus

Il n’existe pas d’évènements indésirables secondaires à la réalisation du test au glucose. Les évènements indésirables sont liés à la prise d’antibiotiques. Le Flagyl peut provoquer les effets indésirables suivants : troubles digestifs, glossite, pancréatite (rare), prurit, éruption cutanée, voire choc anaphylactique, neuropathie, vertige, céphalée, convulsions, confusion, troubles hématologiques. La Noroxine peut entraîner les effets secondaires suivants : des troubles digestifs dont rare pancréatite, des troubles cutanés (rash, érythème, ..), des douleurs musculaires ou articulaires voire rupture tendineuse, troubles neurologiques, troubles psychiques, dyspnée, choc anaphylactique, troubles cardio-vasculaires, troubles hématologiques, atteintes rénale, ou hépatiques.

Ces EIG seront à déclarer selon la même procédure que celle décrite dans le paragraphe 7.2.

# ASPECTS REGLEMENTAIRES ET ETHIQUES

## Balance Bénéfice-Risque

Les effets indésirables des antibiotiques sont relativement rares et cèdent le plus souvent dès l’arrêt du traitement. Ces traitements peuvent améliorer l’incontinence aux gaz qui détériore parfois sévèrement la qualité de vie des patients.

## Soumission au Comité de Protection des Personnes (C.P.P)

Cet essai doit permettre de répondre à la recherche tout en étant conforme à l'éthique médicale. Le protocole, ses amendements éventuels, et toutes les informations sur la recherche sont soumis au C.P.P Nord-Ouest I et à l'Autorité Compétente (AFSSAPS).

En cours d'essai, toute modification de la recherche fera l'objet d'un amendement, qui sera soumis au CPP Nord-Ouest I et à l'Autorité Compétente, avant sa mise en application.

## Consentement écrit des patients et des volontaires sains

Le consentement écrit du patient participant à la recherche sera obtenu avant la réalisation de l'essai. Le patient sera préalablement informé de la nature de l'essai et de ses éventuelles conséquences. Le consentement éclairé est signé par le patient en deux exemplaires, l'un étant remis au patient et l'autre conservé dans le classeur investigateur de l'essai.

Le consentement écrit du volontaire sain participant à la recherche sera obtenu avant la réalisation de l'essai. Le volontaire sain sera préalablement informé de la nature de l'essai et de ses éventuelles conséquences. Le consentement éclairé est signé par le volontaire sain en deux exemplaires, l'un étant remis au volontaire sain et l'autre conservé dans le classeur investigateur de l'essai.

## Information aux directeurs hospitaliers

Les directeurs des centres hospitaliers participant à l'essai seront informés du projet de recherche devant s'y dérouler avant que celui-ci ne soit mis en œuvre.

## Déclaration d'Helsinki, Directive 2001/20/CE

L'essai sera réalisé en conformité avec la dernière version de la Déclaration d'Helsinki (1996) sur les recommandations à l'intention des médecins faisant de la recherche biomédicale chez l'Homme et avec la Directive Européenne 2001/20/CE concernant le rapprochement des dispositions législatives, réglementaires et administratives des Etats membres relatives à l'application de Bonnes Pratiques Cliniques dans la conduite de Recherches Biomédicales.

## Assurance

Elle est souscrite, conformément au Code de la Santé Publique, par le promoteur. Les patients et les volontaires sains participant à l'essai peuvent consulter l'attestation d'assurance.

## Droit d'accès aux données et aux documents source

Conformément aux Bonnes Pratiques Cliniques, l'investigateur autorise l'Attaché de Recherche Clinique mandaté par le promoteur à consulter les dossiers source et s'engage à se rendre disponible pour répondre aux questions soulevées par le promoteur sur la qualité des données recueillies.

L'investigateur autorise également la consultation des données source et accepte la possibilité d'un audit par des personnes autorisées à la demande du promoteur ou des autorités de tutelles.

## Confidentialité

Les résultats sont anonymisés. Les investigateurs s'engagent à ne divulguer ou publier aucun résultat sans l'autorisation du coordinateur. Le traitement informatique des données répondra à la méthodologie MR001 de la C.N.I.L.

## Archivage

Après la fin de l'étude, l'investigateur assurera la conservation des données source des patients, des copies des cahiers d'observation et de la documentation de l’étude contenue dans son dossier pendant au moins 15 ans.

# Publications

Les investigateurs et coordinateurs signeront la version finale du rapport d’essai clinique pour cette étude, indiquant par-là leur accord avec les analyses, les résultats et les conclusions du rapport.

Cette étude fera l’objet d’une publication écrite. Les co-auteurs seront les investigateurs. L’ordre des auteurs sera dicté par le nombre d’inclusions par centre. Le premier auteur sera celui qui aura analysé les données et écrit l’article.

# Références bibliographiques

Attar A, Flourié B, Rambaud JC, Franchisseur C, Ruszniewski P, Bouhnik Y. Antibiotic efficacy in small intestinal bacterial overgrowth-related chronic diarrhea: a cross-over randomized trial. Gastroenterology 1999; 117: 794-797.

Choi YK, Johlin FC, Summers RW, Jackson M, Rao SSC. Fructose intolerance: and under-recognized problem. Am J Gastroenterol. 2003; 98: 1348-53.

Denis P, Bercoff E, Bizien MF, Brocker P, Chassagne P, Lamouliatte H, Leroi AM, Perrigot M, Weber J. Prevalence of anal incontinence in adults. Gastroenterol. Clin. Biol. 1992; 16: 344-350.

Di Stephano M, Malservisi G, Veneto G, Ferrieri A, Corazza GR. Rifaximin versus chlortetracycline in the short-term treatment of small intestinal bacterial overgrowth. Aliment Pharmacol Ther 2000; 14: 551-556.

Di Stefano M, Strocchi A, Malservisi S, Veneto G, Ferrieri A, Corazza R. Non-absorbable antibiotics for managing intestinal gas production and gas-related symptoms. Aliment Pharmacol Ther 2000; 14: 1001-1008.

Di Stephano M, Missanelli A, Mazzocchi S, Corazza GR. Absorbable vs non-absorbable antibiotics in the treatment of small intestine bacterial overgrowth in patients with blind-loop syndrome. Aliment Pharmacol Ther 2005; 21: 985-992.

Fine KD, Schiller LR. AGA technical review on the evaluation and management of chronic diarrhea. Gastroenterology 1999; 116: 1464-86.

Friis H, Bodé S, Rumessen JJ, Gudmand-Hoyer E. Effect of simethicone on lactulose-induced H2 production and gastrointestinal symptoms. Digestion 1991; 49: 227-30.

Gibson PR, Newnham E, Barrett JS, Sheperd SJ, Muir JG. Review article: fructose malabsorption and the bigger picture. Aliment Pharmacol Ther 2007; 25: 349-363.

Johlin FC, Panther M, Kraft N. Dietary fructose intolerance: diet modification can impact self-rated health and symptom control. Nutr Clin Care 2004; 7: 92-7.

Jorge JNM, Wexner SD. Etiology and management of fecal incontinence. Dis Colon Rectum 1993; 36: 77-97.

Leroi AM, Damon H, Faucheron JL, Lehur PA, Siproudhis L, Slim K, Barbieux JP, Barth X, Borie F, Bresler L, Desfourneaux V, Goudet P, Huten N, Lebreton G, Mathieu P, Meurette G, Mathonnet M, Mion F, Orsoni P, Parc Y, Portier G, Rullier E, Sielezneff I, Zerbib F, Michot F. Sacral nerve stimulation in faecal incontinence: position statement based on a collective experience. Colorect Dis 2009; 11: 572-583.

Michot F, Costaglioli B, Leroi AM, Denis P. Artificial anal sphincter in severe fecal incontinence: outcome of prospective experience with 37 patients in one institution. Ann Surg 2003; 237: 52-6.

Mishkin D, Sablauskas L, Yalovsky M, Mishkin S. Frutose and sorbitol malabsorption in ambulatory patients with functional dyspepsia. Dig Dis Sci 1997; 42: 2591-2598.

Pimentel M, Chow EJ, Lin HC. Eradication of small intestinal bacterial overgrowth reduces symptoms of irritable bowel syndrome. Am J Gastroenterol 2000; 95: 3503-3506.

Pimentel M, Chow EJ, Lin HC. Normalization of lactulose breath testing correlates with symptom improvement in irritable bowel syndrome: a double-blind, randomized, placebo-controlled study. Am J Gastroenterol 2003; 98: 412-419.

Pimentel M, Kong Y, Park S. Breath testing to evaluate lactose intolerance in irritable bowel syndrome correlates with lactulose testing and may not reflect true lactose malabsorption. Am. J. Gastroenterol. 2003. 98: 2700-4.

Romagnuolo J, Schiller D, Bailey RJ. Using breath tests wisely in a gastroenterology practice: an evidence-based review of indications and pitfalls in interpretation. Am J Gastroenterol 2002; 97: 1113- 1126.

Rullier E, Zerbib F, Marrel A, Amouretti M, Lehur PA. Validation of the french version of the fecal incontinence quality-of life (FIQL) scale. Gastroenterol. Clin. Biol. 2004; 28: 562-8.

Sheperd SJ, Gibson PR. Fructose malabsorption and symptoms fo irritable bowel syndrome:guidelines for effective dietary management. J Am Diet Assoc 2006; 106: 1631-1639.

Slim K, Bousquet J, Kwiatkowski F, Lescure G, Pezet D, Chipponi J. First validation of the French version of the gastrointestinal quality of life index (GIQLI). Gastroenterol Clin Biol 1999; 23: 25-31.

Quigley EMM, Quera R. Small intestinal bacterial overgrowth: roles of antibiotics, prebiotics and probiotics. Gastroenterology 2006; 130: S78-S90.

Simren M, Stotzer PO. Use and abuse of hydrogen breath tests. Gut 2006; 55: 297-303.

White JG, Hightower NC, Riggs M, Dyck WP. Does activated charcoal relieve gas symptoms ? - a placebo controlled study. Gastroenterology 1991; 100: A261 (Abstract).
