# Supplementary material for: Efficacy of antibiotherapy for treating flatus incontinence associated with small intestinal bacterial overgrowth: A pilot randomized trial
Source: PLoS One. 2017 Aug 1;12(8):e0180835. doi: 10.1371/journal.pone.0180835 (PMC5538639; doi:10.1371/journal.pone.0180835)
Supplement: S4 File — (DOCX) [file pone.0180835.s004.docx]

Statistics : Carbosylane group – total results

Carbosylane group : differences between before and after treatment

Statistics : Metronidazole group – total results

Metronidazole group : Differences between before and after treatment
